# An Evaluation of Urease A Subunit Nanocapsules as a Vaccine in a Mouse Model of *Helicobacter pylori* Infection

**DOI:** 10.3390/vaccines11111652

**Published:** 2023-10-27

**Authors:** Ivana Skakic, Jasmine E. Francis, Chaitali Dekiwadia, Ibukun Aibinu, Mohsina Huq, Aya C. Taki, Anna Walduck, Peter M. Smooker

**Affiliations:** 1 School of Science, RMIT University, 264 Plenty Road, Bundoora, VIC 3083, Australia; ivana.skakic@rmit.edu.au (I.S.); j.ericafrancis@gmail.com (J.E.F.); iaibinu@swin.edu.au (I.A.); m.huq@qu.edu.sa (M.H.); anwalduck@csu.edu.au (A.W.); 2 RMIT Microscopy and Microanalysis Facility, School of Science, RMIT University, Melbourne, VIC 3001, Australia; chaitali.dekiwadia@rmit.edu.au; 3 Department of Health, Science and Community, Swinburne University of Technology, Hawthorn, VIC 3122, Australia; 4 Department of Medical Laboratories, College of Applied Medical Sciences, Qassim University, Buraydah 51452, Saudi Arabia; 5 Faculty of Science, Melbourne Veterinary School, The University of Melbourne, Parkville, VIC 3010, Australia; aya.taki@unimelb.edu.au; 6 Rural Health Research Institute, Charles Sturt University, Orange, NSW 2800, Australia

**Keywords:** *Helicobacter pylori*, vaccination, nanocapsule, silica nanoparticle template, urease alpha subunit

## Abstract

Using removable silica templates, protein nanocapsules comprising the A subunit of *Helicobacter pylori* urease (UreA) were synthesised. The templates were of two sizes, with solid core mesoporous shell (SC/MS) silica templates giving rise to nanocapsules of average diameter 510 nm and mesoporous (MS) silica templates giving rise to nanocapsules of average diameter 47 nm. Both were shown to be highly monodispersed and relatively homogenous in structure. Various combinations of the nanocapsules in formulation were assessed as vaccines in a mouse model of *H. pylori* infection. Immune responses were evaluated and protective efficacy assessed. It was demonstrated that vaccination of mice with the larger nanocapsules combined with an adjuvant was able to significantly reduce colonisation.

## 1. Introduction

*Helicobacter pylori* is a significant human pathogen that infects the gastric mucosa causing gastritis and, in some cases, gastric cancer [1]. *H. pylori* is estimated to infect over 50% of the global population and is most common in areas with poor sanitation [2,3]. Despite the large global burden of disease, *H. pylori* remains without an effective vaccine, despite several pre-clinical vaccines in development [4,5,6,7]. Due to the significant health system burden caused by *H. pylori*, a protective vaccine would be a major step forward in reducing infection and mortality linked to the pathogen. 

Several key antigens have been identified in the quest to develop a vaccine against *H. pylori*. These include urease, catalase, CagA, BabA, VacA, HspA, multivalent epitopes and the FliD protein [8,9]. The urease enzyme was the first bacterial antigenic particle identified for clinical trials of vaccines against *H. pylori* [10].

Urease of *H. pylori* comprises subunits A and B and is an essential factor which contributes to the colonisation of *H. pylori* in the stomach. There is increasing evidence showing *H. pylori* urease to be a key target for therapeutic interventions [9,11]. These include its role in increasing the pH in the stomach, thereby protecting the bacteria from being damaged by the gastric acid [12], its ability to maintain *H. pylori* chronic infection [13], its role as a cause of damage to the gastric mucosal barrier [14] as well as exerting a non-catalytic antioxidant effect which protects *H. pylori* from harmful oxidants [15].

Several studies have been conducted on UreB as a vaccine candidate [7,16], but there is little data on UreA. UreA has been identified as playing a crucial role in the activation of the urease enzyme because of its ability to interact with Hsp60, a protein with an important role in cellular protein homeostasis, while UreB was not identified as being involved in this interaction [17]. UreA-specific CD4^+^ T cells conferred protection to mice in adoptive transfer experiments [18], and recently oral vaccination with UreA expressed on *Bacillus subtilis* spores also elicited protective responses to *H. pylori* challenge [19].

While protein-based vaccine formulations are generally safe and cost-efficient to produce, protein alone can be poorly immunogenic and requires an adjuvant to stimulate protective immune responses [20,21]. Previous studies on particulate-based antigens indicate that an antigen in particulate form can improve vaccine immunogenicity through improved antigen protection and sustained antigen release, which render an adjuvant-like effect, as reviewed by Taki and Smooker [22]. As discussed in this review, it is well known that the sizing of nanoparticles can determine the pathway of uptake and influence the resulting immune response.

Furthermore, in a previous study [23], we identified that monodispersed silica nanoparticles have unique properties which make them suitable for synthesising antigen-based nanocapsules. We demonstrated that nanocapsules with diameters of 40 and 550 nm could be readily taken up by dendritic cells (DCs) and presented by the cross-presentation pathway [23,24]. Such particles are derived by infusing protein into a porous silica “template”, cross-linking the protein and subsequently removing the template. The advantage that these nanocapsules may have over other formulations is that as the template is removed, the capsules are only composed of pure, cross-linked protein, which may result in a high antigenic dose per unit of capsule and also alleviate the toxicity concerns of some systems (reviewed in [22]). Lastly, this is a broadly applicable “platform” technology, as theoretically any pathogen antigen could be used to form nanocapsules.

Antigenic nanocapsules are therefore an important emerging vaccine approach warranting further investigation. In this report we describe the formation of such particles (termed nanocapsules) from the recombinant urease A subunit of *H. pylori* and evaluate their vaccine potential in a mouse model of *H. pylori* infection.

## 2. Materials and Methods

### 2.1. Preparation of Silica Templates

Fabrication of the solid core/mesoporous shell (SC/MS) and mesoporous shell (MS) silica templates was undertaken based on the method reported by Büchel and colleagues [25] and elaborated in our previous publications [23,24]. Two different templates were prepared. The first was an SC particle with a surrounding MS shell (SC/MS). These were designed to be approximately 500 nm in diameter. The second was an MS particle with a designed diameter of approximately 50 nm.

The solid silica particle core (SC) was prepared based on a modified version of the Stöber method, which involves the hydrolysis and condensation of tetraethoxysilane (TEOS) in ethanol, MQH_2_O and ammonia solution. More specifically, 37 mL of absolute ethanol (Merck, Darmstadt, Germany), 5 mL of MQH_2_O (Millipore, Burlington, MA, USA) and 4.2 mL of 32% ammonium hydroxide (Merck, Darmstadt, Germany) were combined in an Erlenmeyer flask and stirred vigorously at room temperature. Once the temperature of the solution had stabilised, 2.8 mL of TEOS (Sigma-Aldrich, Darmstadt, Germany) was added promptly and vortexed for 10 sec to ensure homogeneity of the nanoparticles. After vortexing, the solution was kept still at room temperature for 1 h to allow the reaction to proceed.

The SC/MS nanoparticles were prepared based on the method reported by Büchel and colleagues [25], which involves particle synthesis by sol-gel coating of a mesoporous shell (MS) on a pre-formed solid silica particle, as detailed by Skakic et al. [24]. To a solution containing SC particles, a mixture of 2.35 mL TEOS and 0.5 mL 91.6% TMS (Sigma-Aldrich, Darmstadt, Germany) was added slowly over a 20 min period while stirring. The solution was then incubated for 2.5 h at room temperature, allowing the formation of the MS. Following the incubation, the solution was washed three times with ethanol at 5000× *g* for 2 min. The SC/MS particles were then allowed to dry on a Petri dish at room temperature overnight to completely remove ethanol. The porogen TMS was removed through calcination by heating at 550 °C for 6 h. Once calcined, the SC/MS particles were kept dry in a polypropylene tube—in this condition, the SC/MS particles could theoretically be stored indefinitely.

MS silica nanoparticle templates were prepared based on the method reported by Möller [26] and in detail by Skakic et al. [24]. A stock solution of 64 mL MQH_2_O, 10.5 mL ethanol and 10.4 mL 25% cetyltrimethylammonium chloride (CTAC) (Sigma-Aldrich, Darmstadt, Germany) solution was prepared by stirring for 10 min at room temperature. To this solution, 4.125 mL triethanolamine (TEA) (Sigma-Aldrich, Darmstadt, Germany) was added slowly and stirred until dissolved. Next, 20 mL of this solution was heated to 60 °C and subsequently 1.454 mL of TEOS was added dropwise while stirring, then left to cool. Templates were collected at 47,800× *g* for 20 min and then washed twice with distilled H_2_O, with sonication between washes. Particles were left to dry in a petri dish overnight at room temperature, and subsequently the porogen TMS was removed through calcination by heating at 550 °C for 6 h. Calcined MS particles were then kept dry in a polypropylene tube.

### 2.2. Preparation of Recombinant UreA

The synthesis and cloning of the UreA gene into the pRSETa plasmid was conducted externally (Life Technologies, Melbourne, Australia) according to the UreA sequence (UniProt ID:P14916). The resultant plasmid was termed pRSETa_A. The plasmid was transformed into *Escherichia coli* strain BL21(DE3) for protein expression by standard procedures. The purified recombinant UreA was buffer exchanged, concentrated and pooled. All recombinant UreA that was used for synthesis of nanocapsules in this study underwent endotoxin removal (Thermo Fisher Scientific, Waltham, MA, USA). The concentration was estimated by Bradford assay. The purity of final UreA sample was assessed by sodium dodecyl sulphate polyacrylamide gel electrophoresis (SDS-PAGE) and Western blotting as described previously [24].

### 2.3. Nanocapsule Synthesis and Characterisation Protocols

UreA SC/MS and MS nanocapsules were prepared by the incubation of SC/MS and MS silica templates with purified recombinant UreA at a 3:1 *w*/*w* ratio in PBS (sodium chloride 0.8%, potassium chloride 0.02%, disodium hydrogen phosphate 0.115%, potassium dihydrogen phosphate 0.02%, pH 7.4) overnight at 4 °C on an end-over-end suspension mixer. Following infiltration, excess protein was removed by PBS washes and cross-linking was undertaken using 5% (*w*/*v*) glutaraldehyde (Sigma-Aldrich, Darmstadt, Germany) for 2 h at 4 °C on an end-over-end suspension mixer. Excess GA was removed by PBS washes, and the silica template was removed by 80% (*v*/*v*) of 2 M hydrofluoric acid treatment and the remaining UreA-based nanocapsules were resuspended in sterile PBS and stored at 4 °C.

UreA nanocapsules in this study were characterised as follows: The structural characterisation of UreA nanocapsules was assessed via Dynamic Light Scattering (DLS) and Transmission Electron Microscopy (TEM) as previously described [22]. The loading capacity and loading efficiency was calculated to quantify the amount of UreA nanocapsules prepared in a vaccine and administered to an animal model. To assess the amount of UreA infiltration into the templates, the amount of protein was measured before and after the infiltration process. Using the equation below [27], the loading capacity (LC) and loading efficiency (LE) of protein infiltration were calculated.

LC (mg of protein/g of template = (total amount of protein) − (free amount of protein)/

Weight of template

%LE = (total amount of protein) − (free amount of protein in washes) × 100/

(total amount of protein)

### 2.4. Animal Trial

All animal experiments were undertaken with approval from the RMIT Animal Ethics Committee under the Animal Ethics Committee approval number AEC1723. All experiments and procedures were undertaken following approved animal handling and experimental protocols.

Six-week-old female *Helicobacter*-free C57BL/6 mice were purchased from the Animal Resource Centre, Canningvale, Western Australia. A total of 96 mice were used in the trial, with 8 mice randomly assigned to each group as shown in Table 1. Mice were fed a sterile standard diet, had access to water ad libitum and were housed under specific pathogen-free conditions.

Mice were vaccinated once (Group A, live attenuated *Salmonella* strain expressing UreA and UreB proteins), or twice (Groups B–L) two weeks apart as indicated in Table 1. Mice in Group A were vaccinated with a recombinant attenuated *Salmonella enterica var* Typhimurium (strain SL3261pYZ97) previously shown to reduce colonisation in mice [28]. This therefore served as the positive control. Nanocapsule formulations or recombinant protein were suspended in sterile saline and emulsified in TiterMax Gold adjuvant (Sigma Aldrich) in a 1:1 water in oil emulsion, as recommended by the manufacturer. A dose of 100 µL of each emulsion was injected.

To characterise the immunogenicity of the vaccines prior to the live *H. pylori* challenge, blood samples were collected 2 weeks following the second vaccination. Four mice were randomly selected from each group, and a total of 100 µL of blood was collected from each mouse via saphenous vein puncture.

### 2.5. H. pylori Challenge

Two weeks after the second vaccination, mice were challenged orally in 0.1 mL with 10^7^ CFU *H. pylori* Sydney strain SS1 [29] by oral gavage [30]. The SS1 strain has been shown to induce gastritis, but infection is asymptomatic in wild-type mice. Mice were killed three weeks after challenge with *H. pylori*.

### 2.6. Serum, Blood and Tissue Collection

To characterise antibody responses after the oral *H. pylori* challenge, serum, blood and tissue was collected from mice at the termination of the experiment, three weeks after oral challenge. Blood was collected by cardiac puncture, and tissue harvest included mesenteric lymph nodes (MLN), stomach and spleen. Three quarters of the stomach of was used for flow cytometry analysis, and one quarter of the stomach were homogenised and DNA was extracted to determine the level of colonisation.

### 2.7. Statistical Analysis

GraphPad Prism software (Version 8, GraphPad Software, Graphpad.com) was used to perform the statistical analysis for this study. Statistical variables, including mean, standard deviation and standard error were calculated for all data. Comparisons between groups were performed by one-way ANOVA followed by a Kruskal–Wallis test using Dunn’s multiple comparison test to determine significant differences between groups. A *p*-value of 0.05 was used to determine significance.

### 2.8. Immunoassay Protocols

For pre-challenge antibody level analysis, IgG, IgG1 and IgG2c data was compared to the PBS naïve control (Group C). For endpoint antibody levels of IgG, IgG1 and IgG2c, data was compared to the PBS control (Group B) as this group had been challenged with *H. pylori* making it the most appropriate comparison to experimental vaccine groups which had also undergone *H. pylori* challenge.

### 2.9. Measurement of Humoral Response by Serum Antibody Detection—Indirect ELISA

To determine specific titres of whole IgG and subtypes IgG1 and IgG2c, blood was collected from mice after the second vaccination, pre-challenge and at the termination of the trial. Blood was centrifuged at 14,000× *g* for 3 min, and the serum fraction was collected and stored at 20 °C until use.

ELISA assays were performed in 96-well plates (Nunc, Roskilde, Denmark), and mouse sera was assayed in duplicate across serial doubling dilutions at a starting dilution of 1:100 in PBS. Wells were coated with 100 µL of 2 µg/mL recombinant UreA in coating buffer overnight at 4 °C. Coating buffer and unbound antigen was removed, and plates were washed 3× with ELISA wash buffer (PBS with 2% Tween20 (Sigma-Aldrich, Darmstadt, Germany) before blocking with ELISA blocking buffer for 2 h at 37 °C. Blocking buffer was subsequently removed and mouse antisera diluted 1:100 in PBS/Tween with skim milk (1% *w*/*v*) was added and serially diluted across the ELISA plate. Plates were incubated for 2 h at 37 °C before being washed 3× to remove unbound antibody and patted dry. Detection antibody was diluted to 1:10,000 in PBS/Tween with skim milk (1% *w*/*v*) and 100 µL was added and plates incubated for 1 h at 37 °C. Plates were washed 3× with PBS/Tween and once with PBS alone before the addition of 100 µL substrate to each well, which was allowed to develop for up to 30 min per plate and stopped with 50 µL 2 M HCL (Merck, Darmstadt, Germany). Absorbance was measured at 450 nm using the POLARstar Omega Plate Reader (Labtech, Ortenberg, Germany).

### 2.10. Immune Cell Population Quantification by FACS

Mesenteric lymph nodes and spleens were harvested from mice at the termination of the experiment and tissues from each mouse were individually crushed through a 70 µm cell strainer (Corning, Corning, NY, USA) into ice cold PBS with 2% FBS to collect single-cell suspensions for FACS staining and immune cell population assays. Cells were counted by trypan blue exclusion and approximately 10^6^ cells per sample were aliquoted into a 96-well plate for FACS staining.

Gastric lymphocytes were isolated using the optimised method described by Ng and Sutton [31]. The isolated cell suspension was counted and adjusted to a concentration of approximately 10^6^ cells/mL prior to staining.

Prior to antibody staining, live/dead staining was performed using the Zombie Aqua™ Fixable Viability Kit (Biolegend, San Diego, CA, USA) as recommended by the manufacturer, then pelleted by centrifugation (1500 rpm, 3 min) and resuspended in ice cold FACS buffer (3% *v*/*v* FBS in PBS). Cells were incubated with FC block™ (BD Biosciences, USA) at 4 μg/mL in 50 μL FACS buffer for 15 min and washed as described above. The following antibodies were used for surface staining: CD3 (145-2C11), CD45 (30-F11), CD4^+^ (GK1.5), CD8^+^ (53.6.7), CD11b^+^ (M1/70) and Ly6G^+^ (1A8) (all from BD Biosciences, Franklin Lakes, NJ, USA). Cells were stained for 30 min on ice. After surface staining, cells were washed and then permeabilised using the Fix/Perm kit (BD Biosciences, Franklin Lakes, NJ, USA) according to the manufacturer’s instructions. Cells were incubated with anti-mouse IFNγ antibody for 30 min on ice. After 3 washes, cells were resuspended in PBS for analysis using a BD Canto II flow cytometer (BD Biosciences, Franklin Lakes, NJ, USA) and analysed using FlowJo software (Version 8.5, BD Biosciences, Franklin Lakes, NJ, USA). A minimum of 10,000 cells per sample were collected. Cells were gated to exclude aggregates and dead cells. Populations through the lymphocyte gate were defined as CD4^+^ T cells (CD45^+^/CD3^+^/CD4^+^), CD8^+^ T cells (CD45^+^/CD3^+^/CD8^+^), Macrophages (CD45^+^/CD3^−^/CDllb^+^) and Neutrophils (CD45^+^/CD3^−^/CD11b^+^/Ly6G^+^).

### 2.11. H. pylori Stomach DNA Analysis

Mouse stomach homogenates were centrifuged at 14,000× *g* for 1 min and the resulting supernatant was discarded. DNA was extracted from homogenates using the protocol described by Zangala [32] modified for 2 mL volume with the addition of treatment with lysozyme 5 mg/mL for 30 min. DNA was precipitated using ethanol and resuspended in sterile TE Buffer. The concentration of DNA in each sample was determined using a Nanodrop spectrophotometer (Thermo Fisher Scientific, Waltham, MA, USA).

### 2.12. H. pylori Burdens Analysis by qPCR

Stomach DNA samples were analysed by Taqman qPCR to detect the presence of *H. pylori* 16S RNA and quantify burden against *H. pylori* DNA standards ranging from 0.0001–100 ng. Samples were amplified in 20 µL PCR reactions using Bioline SENSIFast probe No-ROX Mastermix (Meridian Bioscience, Lukenwalde, Germany), containing 300 ng DNA, 0.4 μM forward and reverse primers (Table 2) and 0.1 μM probe. Conditions for qPCR were as follows: 95 °C, 10 min, 45 cycles, 95 °C 10 s, 60 °C, threshold. DNA extracted from PBS naïve control stomachs was used to set the baseline above which a signal threshold was applied. Results were expressed at *H. pylori* genomes/g tissue.

## 3. Results

### 3.1. Protein Expression and Characterisation

**Figure 1:**
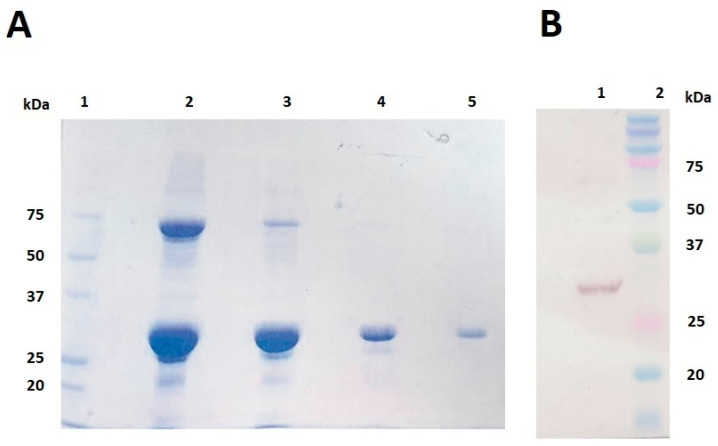
(**A**) SDS-PAGE gel of recombinant UreA following endotoxin removal and final concentration. Lane 1: Precision Plus Protein™ Unstained Protein Standards; Lane 2: neat sample; Lane 3: ½ dilution of sample; Lane 4: 1/10 dilution of sample; Lane 5: estimated 1 µg of sample. (**B**) Immunoblot of recombinant UreA following endotoxin removal and final concentration. Lane 1: estimated 1 µg of sample; Lane 2: Precision Plus Protein™ Kaleidoscope™ Prestained Protein Standards.

Recombinant UreA was expressed as a soluble protein in *E. coli* BL21(DE3), purified by IMAC, buffer exchanged and concentrated before undergoing endotoxin removal. It was then analysed by SDS-PAGE. Figure 1A shows UreA was present at the expected size of 31 kDa, and the sample indicated the presence of some other proteins when viewed in the overloaded lanes (lanes 3 and 5). Presence of UreA was confirmed by probing for the 6His-tag attached at the N-terminal of the sequence via Western immunoblotting at the expected size of 31 kDa (Figure 1B). The main species identified in the Coomassie stained gel at around 60 kDa is the expected size for a dimer, which is consistent with it also being detected in the Western blot.

### 3.2. Protein Loading of Silica Templates

The SC/MS template was infiltrated with up to 90 mg of UreA protein/g of nanoparticle template, and the MS template was infiltrated with up to 140 mg of UreA protein/g of nanoparticle template. The loading efficiencies were 27% for the SC/MS template, and 42% for the MS template.

### 3.3. Characterisation of Silica Templates and UreA Nanocapsules by Transmission Electron Microscopy (TEM)

**Figure 2:**
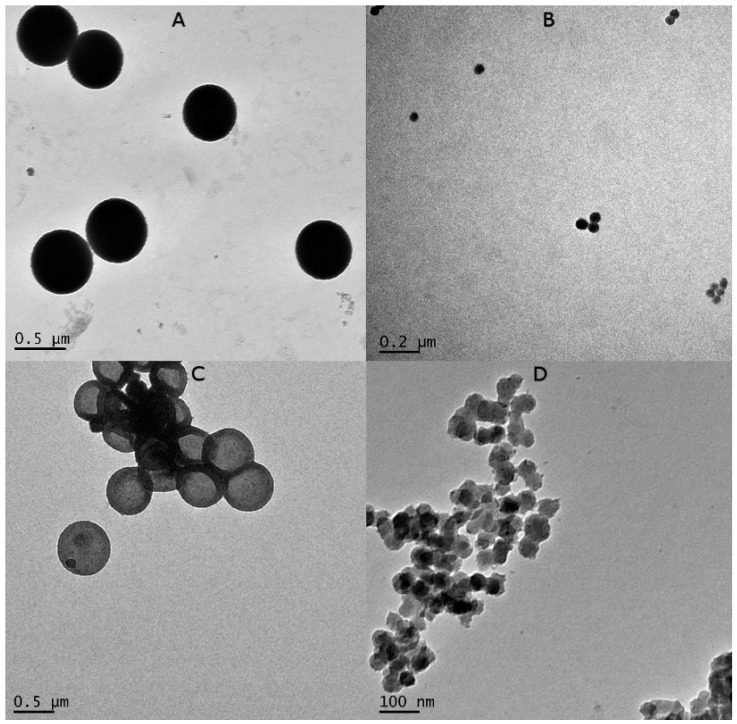
TEM image analysis of SC/MS silica templates (**A**) and MS templates (**B**). (**C**,**D**) show SC/MS UreA-based nanocapsules and MS UreA-based nanocapsules, respectively. Imaging revealed that templates and nanocapsules were homogenous in size and morphology within each size range.

Silica templates and UreA nanocapsules were loaded onto holey carbon grids (ProSciTech, Townsville, Australia) and stained with phosphotungstic acid (Sigma-Aldrich, Darmstadt, Germany) before being visualised and imaged by JEOL1010 TEM (JEOL, Tokyo, Japan) at 100 kV accelerating voltage. TEM analysis revealed that SC/MS and MS particle templates were spherical and homogenous in structure. The average diameter of synthesised SC/MS particles depicted in Figure 2A was 523.75 ± 2.56 nm, and the average diameter of the MS particles depicted in Figure 2B was 53.90 ± 3.8 nm.

TEM micrographs of SC/MS and MS UreA nanocapsules revealed capsules which were homogenous in both size and morphology. SC/MS UreA nanocapsules shown in Figure 2C were found to be homogenous in size, with creasing indicating a hollow-core capsule with an average diameter of 510.3 ± 18.21 nm. The MS UreA nanocapsules shown in Figure 2D were also found to be homogenous in size and structure, with some predisposition to capsule aggregation, and had an average diameter of 47.3 ± 9.14 nm.

### 3.4. Size and Zeta Potential Characterisation by Dynamic Light Scattering (DLS)

**Figure 3:**
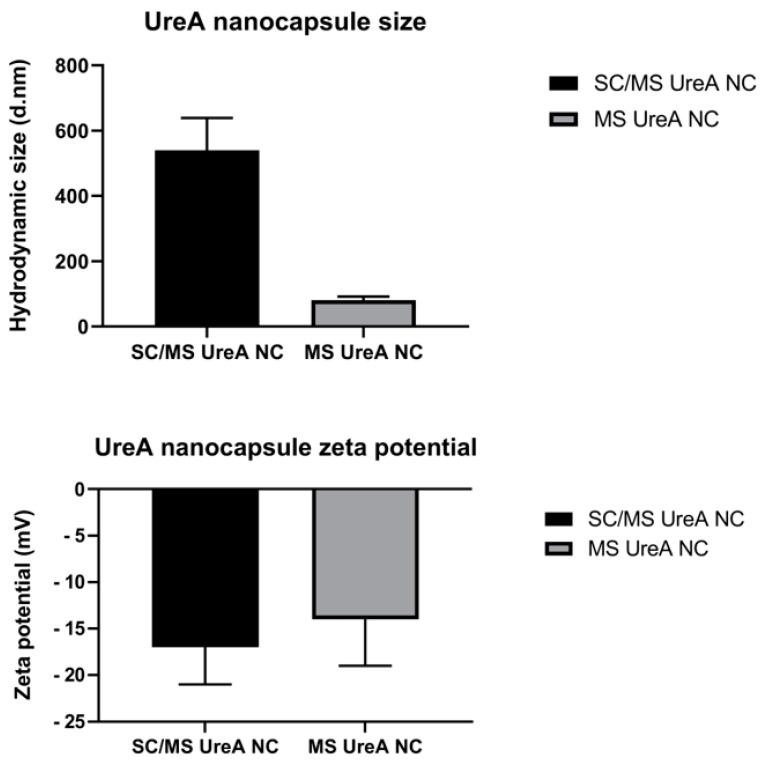
DLS analysis of nanocapsules. This indicates SC/MS UreA nanocapsules were well dispersed. MS UreA nanocapsules showed some evidence of aggregation. Both capsule size ranges possessed a negative zeta potential.

Both SC/MS and MS UreA nanocapsules were characterised by DLS using the Zetasizer Nano ZS (Malvern Instruments, London, UK), assessing nanocapsule size and zeta potential. Figure 3 shows the size of SC/MS and MS nanocapsules measured by DLS. The average size of the SC/MS nanocapsules was measured as 539.5 nm, and the average size of the MS nanocapsules was 82.4 nm. This, coupled with the TEM imaging, does indicate some aggregation of the MS nanocapsules. The lower panel shows the zeta potential of the SC/MS and MS nanocapsules measured by DLS. Analysis revealed that capsules had a negative zeta potential within each size range. The average zeta potential of the SC/MS capsules was indicated at −17 mV and that of the MS capsules at −14 mV.

### 3.5. Levels of Antigen-Specific IgG, IgG1 and IgG2c Antibodies

Antigen-specific IgG titres for individual mice were determined by indirect ELISA. The titre cut-off point was determined based on the mean and three standard deviations from the negative control readings. IgG, IgG1 and IgG2c levels were expressed as a logarithm of the titre.

**Figure 4:**
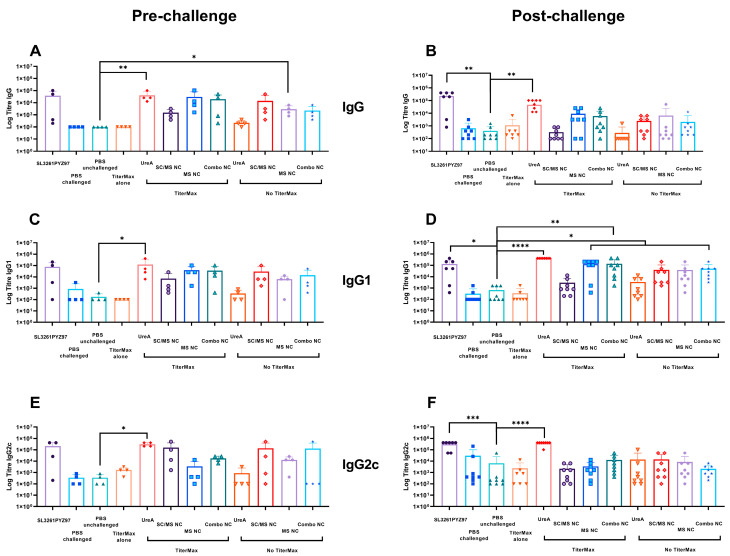
Titre of immunoglobulins in immunised mice pre-challenge (left) and post-challenge (right) with asymptomatic *H. pylori.* (**A**,**B**) IgG. (**C**,**D**) IgG1. (**E**,**F**) IgG2c. See Table 1 for group details. Note that the third group (PBS-Naïve) was not challenged. * *p* ≤ 0.05, ** *p* ≤ 0.01, *** *p* ≤ 0.001 and **** *p* ≤ 0.0001.

Mice were immunised as listed in Table 1. Serum samples were collected from individual mice prior to *H. pylori* infection (termed ‘pre-challenge’) and after challenge at the endpoint of the trial (termed ‘post-challenge’). As seen in Figure 4, Endpoint titres were demonstrably elevated in all groups following *H. pylori* challenge compared to the pre-challenge titres (comparing Figure 4A,C,E and Figure 4B,D,F, respectively). Serum analysis of whole IgG from individual mice pre-challenge is shown in Figure 4A. Mouse sera assayed for whole IgG showed significant levels of IgG in the soluble UreA with TiterMax group, significantly higher than the PBS naïve control group (*p* ≤ 0.004). Significant levels of IgG were also seen in the MS UreA nanocapsules with TiterMax group, which was significantly higher compared to the PBS naïve control group (*p* ≤ 0.02). No significant difference was detected between the SL3261pYZ97 control or remaining nanocapsule vaccine groups compared to the PBS control for whole IgG pre-challenge sera.

The level of specific IgG1 and IgG2c in individual mice sera was determined and the mean titre was calculated as shown (Figure 4). Mouse sera analysed for IgG1 subtype antibodies showed significant levels of IgG1 in the soluble UreA with TiterMax group compared to the PBS naïve control (*p* ≤ 0.02), indicative of a potential Th2 response to the soluble protein prime and boost (Figure 4C). No significant difference was detected between the SL3261pYZ97 control or the nanocapsule vaccine groups compared to the PBS control for IgG1 pre-challenge sera (Figure 4C). As an indicator of Th1 responses, mouse sera were assayed for IgG2c responses (Figure 4E), and data showed significant levels of IgG2c in the soluble UreA with TiterMax compared to the PBS naïve control (*p* = 0.02). No significant difference was detected between the SL3261pYZ97 control or the nanocapsule vaccine groups compared to the PBS control for IgG2c pre-challenge sera (Figure 4D).

Serum samples were collected three weeks following challenge with *H. pylori* and tested for humoral immune responses by detecting the levels of UreA-specific IgG antibodies. The endpoint IgG, IgG1 and IgG2c analysis was compared to the PBS control group as this group had been challenged with *H. pylori* but received no vaccine formulation and was therefore deemed the most appropriate comparison to assess vaccine efficacy in this study. Endpoint titres of IgG antibody shown in Figure 4B indicated significant antibody levels in sera from mice vaccinated with the SL3261pYZ97 control compared to the PBS control (*p* ≤ 0.006). The soluble UreA with TiterMax group also showed significant titre levels compared to the PBS control (*p* ≤ 0.003).

Significant levels of UreA-specific IgG1 antibodies (Figure 4D) were highest in endpoint sera from mice which received the soluble UreA with TiterMax compared to PBS alone (*p* ≤ 0.0001). Sera from mice vaccinated with combo nanocapsule with TiterMax showed high levels of IgG1 antibody compared to the control (*p* ≤ 0.001). Mice vaccinated with the SL3261pYZ97 control, as well as those vaccinated with the combo nanocapsule vaccine and MS nanocapsule with TiterMax vaccine, all had significantly higher endpoint titres compared to the PBS control (*p* ≤ 0.005). Notably, the nanocapsule groups without the addition of TiterMax adjuvant also showed significantly higher IgG1 antibody levels compared to the control; ie: the SC/MS nanocapsule group compared to the PBS group (*p* ≤ 0.03), and the MS nanocapsule group compared to the PBS group (*p* ≤ 0.04).

Endpoint mouse sera assayed for IgG2c antibody subtype (Figure 4F) showed significant levels of IgG2c in the soluble UreA with TiterMax group (*p* ≤ 0.003) and the SL3261pYZ97 control sera (*p* ≤ 0.009) compared to the PBS control. No other groups showed significant changes in IgG2c antibody levels.

### 3.6. Determination of H. pylori Burdens

*H. pylori* burden was determined by qPCR specific for the 16sRNA gene. Statistical significance was determined by Mann Whitney test with Dunn’s multiple comparison test.

**Figure 5:**
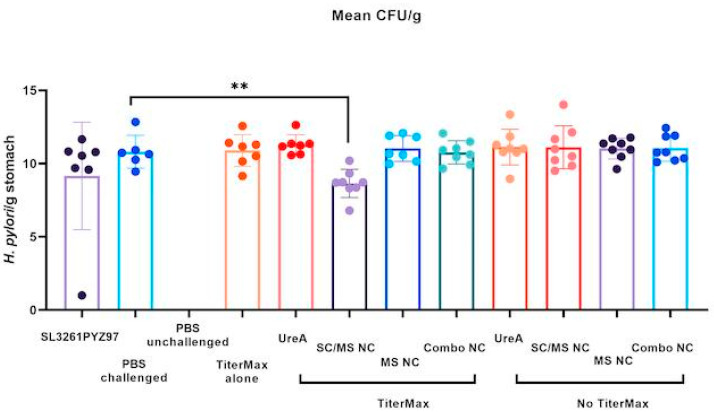
qPCR detection of *H. pylori* burdens in stomachs of vaccinated mice. Mice vaccinated with SC/MS nanocapsules with TiterMax had significantly reduced levels of gastric *H. pylori* burden compared to the infected control (** *p* ≤ 0.01, one-tailed). No significant difference was detected between mice vaccinated with any other vaccine groups.

As depicted in Figure 5, mice vaccinated with SC/MS nanocapsules with TiterMax had significantly reduced levels of gastric *H. pylori* burden compared to the infected control (*p* ≤ 0.01, one-tailed), representing a 1.3 fold reduction log10, which translates to a 177-fold reduction in total bacterial numbers (not significant in a two-tailed test). This reduction is less than the 1.5–3 log reductions (4.5 × 10^3^–1.8 × 10^7^ fold reduction in bacterial numbers) reported previously for the SL3261pYZ97 [30,34] and orally and intranasally delivered bacterial protein vaccines [35]. In this study, the SL3261pYZ97 control group had a mean fold reduction of 1.2 log10; this was not significant compared to the PBS control group. This result was unexpected, given previous reports discussed above. This reason for this is unknown, but may be attributed to the dose of SL3261pYZ97 having been lower than calculated. Compared to unvaccinated controls, there were no significant reductions in *H. pylori* burden observed in either of the soluble UreA groups, MS nanocapsule groups, combo nanocapsule groups or the SC/MS nanocapsules without TiterMax.

### 3.7. Analysis of Immune Cell Populations from Mouse Tissue

**Figure 6:**
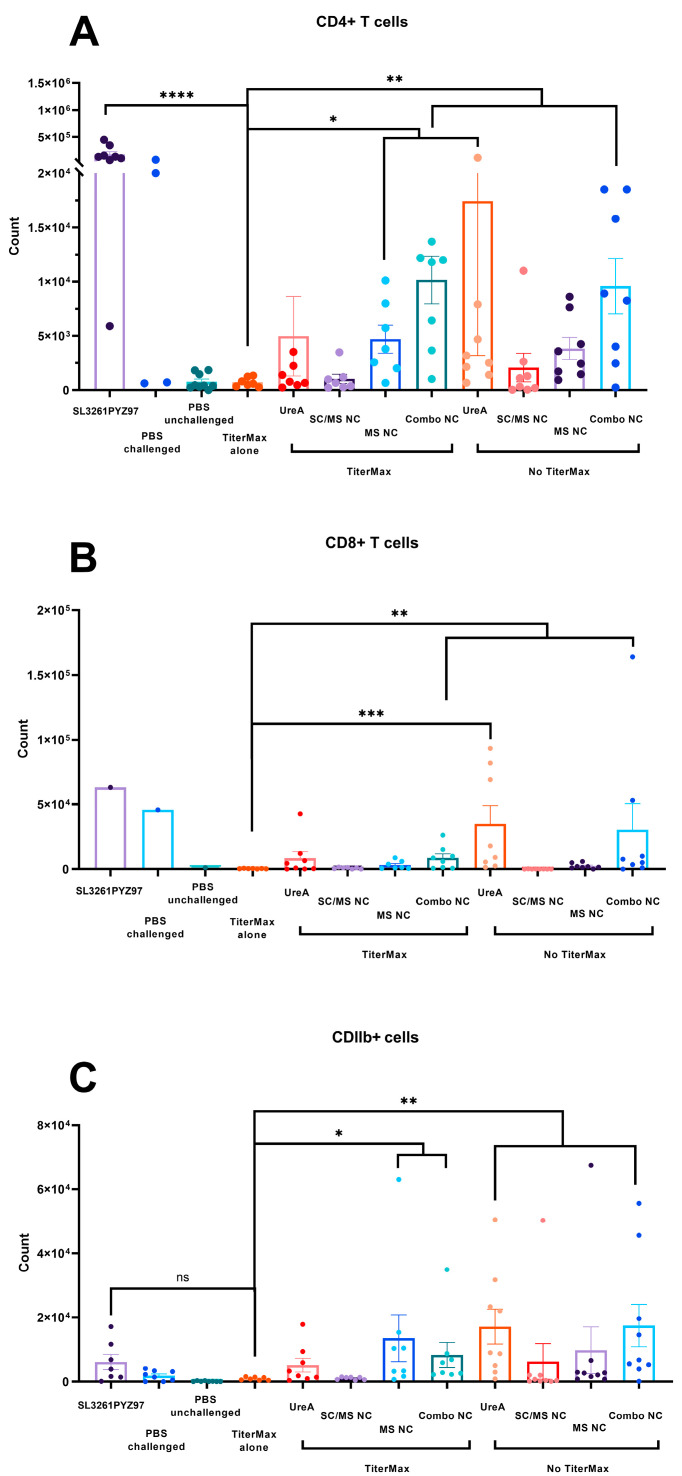
Analysis of infiltrating immune cells in stomach tissue of vaccinated mice. (**A**–**C**) Infiltration of CD4^+^, CD8^+^ and CDIIIb^+^ cells, respectively. (Note that cells values for SL3261pYZ97 vaccinated mice are pools of cells from 5 mice, SEM not possible) * *p* ≤ 0.05, ** *p* ≤ 0.01, *** *p* ≤ 0.001 and **** *p* ≤ 0.0001.

Infiltrating immune cell populations in mouse stomach tissue were stained for relevant surface markers and analysed by flow cytometry. Statistical significance was determined by Kruskal–Wallis test with Dunn’s multiple comparison test and significance was represented as shown in Figure 6, determined using GraphPad prism software version 8. For each experiment, *n* = 7–8 individuals per group.

In samples with low cell counts from individual mice, up to 7 mice were pooled for some analyses. Therefore, the TiterMax only group was determined as the most appropriate to use as a comparison control. Statistical analysis for CD4^+^ T cells, CD8^+^ T cells and CD11b^+^ cells was performed and compared to TiterMax only control and presented (Figure 6A–C). A minimum of 10,000 cells per sample were collected.

Mice challenged with *H. pylori* had increased numbers of infiltrating CD4^+^ T cells compared to the non-challenged control. Use of TiterMax adjuvant did not enhance CD4^+^ T cells in combination with UreA protein or nanocapsule formulations. MS/NC and Combination NC vaccinated mice has significantly increased levels of CD4^+^ T cells compared to the control. SL2361pYZ97 vaccinated mice had high levels of infiltrating CD4^+^, consistent with previous reports [30]. Similarly, combo NC elicited significant increases of CD8^+^ T cells, but this did not differ from levels induced by *H. pylori* challenge alone or the Salmonella vaccine (Figure 6B). Numbers of infiltrating CD11b^+^ cells were low, and only significantly increased in soluble UreA and combination nanocapsule vaccinated mice.

## 4. Discussion

In this study, UreA-based nanocapsules were synthesised as a novel vaccine against *H. pylori* and tested for efficacy in mice using a live *H. pylori* challenge model. The successful construction of two different-sized templates and the resulting nanocapsules demonstrates the flexibility of this method. Theoretically, any protein antigen could be used as the basis for such particles and used as a vaccine. We have previously reported on the construction of nanocapsules using the ovalbumin protein [23] and have also constructed nanocapsules using other proteins (unpublished data). The particles we constructed here were used as antigens in a vaccine trial using a mouse model of *H.* pylori infection. To our knowledge, this is the first report of the use of these types of protein-only nanocapsules as vaccine antigens. The experimental groups in the study included SC/MS capsules, MS nanocapsules and a combination of both types of nanocapsules. Each of these experimental groups had a comparative group that included TiterMax adjuvant. An attenuated *Salmonella enterica* serovar Typhimurium (*S. typhimurium* SL3261pYZ97) expressing UreA and UreB subunits was used as a positive control vaccine, which has been shown to confer a 1–2 log reduction in *H. pylori* colonisation and is therefore considered protective [28]. Interestingly, in this trial, it did not confer significant protection. While this result is unexpected, it may simply represent biological variation, as only one Salmonella-vaccinated animal responded. This result does not affect our interpretation of the efficacy of the nanocapsule formulations in the correct study, as the primary aim of the study was to test the nanocapsules.

Synthesised SC/MS and MS templates and nanocapsules were visualised by TEM which revealed homogenous morphology within each sample. Analysis of the SC/MS nanocapsules revealed an average diameter of approximately 500 nm, with particles appearing flattened and deflated, indicative of the hollow-core structure, as expected from previous characterisation studies [23]. Particle size results were validated by DLS analysis, which indicated an average diameter of 540 nm for the SC/MS nanocapsules. TEM analysis of the MS nanocapsules indicated an average diameter of approximately 50 nm with a homogenous and spherical morphology and a highly porous structure evident from TEM imaging. However, DLS analysis for MS nanocapsules determined that the average diameter was 82.4 nm, likely a result of a degree of particle aggregation. DLS analysis indicated that SC/MS nanocapsules and MS nanocapsules possessed negative zeta potentials of −17 mV and −14 mV, respectively. This may account for the particle aggregation observed with the MS nanocapsules, as zeta potential measures electrostatic attraction or repulsion between particles and is an effective indicator for the stability of dispersions. Higher zeta potential tends to indicate highly charged particles which prevents particle aggregation due to their electrostatic repulsion from one another. Conversely, if zeta potential is low, then there is an increased likelihood that attraction of particles will be more dominant, resulting in particle aggregation [36,37].

The strain used to challenge vaccinated mice in this study is the *H. pylori* Sydney Strain 1 (SS1), which was developed as a model strain of *H. pylori* demonstrating high colonising ability [29] and is considered an effective challenge model for *H. pylori* studies in C57BL/6 mice. The results of this study infer successful in vivo antigen processing and presentation of the UreA protein in soluble and nanocapsule-based formulations. Total IgG and antibody subtypes IgG and IgG2c were measured to determine humoral responses to the UreA nanocapsule vaccines. An IgG1 antibody response is classically stimulated by the Th2-dependant cytokine IL-4, whereas a Th1-biased immune response generated by INFγ is indicated by a IgG2a antibody response [38]. The IgG2a isotype is not expressed in mouse strains with the Igh1-b allele, which includes the C57BL/6 mouse strain used here, and instead this strain expresses the IgG2c isotype [39] which was measured in this study as an indicator of a Th1 immune response.

In this study, IgG antibodies were measured as an indication of a humoral response to vaccination. However, studies have indicated that essential components of successful vaccination against *H. pylori* infection involve a mucosal immune response, with CD4^+^ T cells being essential for gastric clearance [40]. Antibodies are not considered essential for protection against *H. pylori*, and may potentially be disruptive to effective protection [41]. Furthermore, infection with *H. pylori* Sydney strain 1 (SS1) in mice results in low levels of mucosal IgA antibody responses. A cross-sectional study in patients undergoing upper gastrointestinal endoscopy found that severity of gastritis correlated with IgG antibody titre against *H. pylori*, concluding that IgG titre was significantly higher in patients harbouring cytotoxin-associated gene (*cagA*)-positive *H. pylori* strains [42]. IgG levels may therefore be useful as a marker for severity of infection with certain *H. pylori* strains.

While not essential for protection against *H. pylori*, antibodies are protective in many disease models, and investigation of antibody responses sheds light on the immunogenicity of UreA nanocapsules as a vaccine model as well as the role of particle size and structure on immunogenicity in a mouse model. Characterisation of antibody levels prior to the *H. pylori* challenge found no significant IgG1 or IgG2c responses from any of the groups vaccinated with nanocapsules. However, following *H. pylori* challenge, mice vaccinated with MS nanocapsules with TiterMax as well as both of the combo nanocapsule groups showed significant IgG1 antibody responses and no IgG2c response, indicating a Th2-biased immune response [38]. This trend towards a Th2-biased response suggests that these nanocapsules generated a strong humoral response. Notably, neither of the SC/MS nanocapsule groups generated significant antibody responses, whilst the MS nanocapsules with TiterMax and both the combo nanocapsule groups did generate significant IgG1 antibody levels. This may suggest that the antibody response was related to nanocapsule size. Previous studies have shown that cell uptake of particles is size-dependant [43]; the heterogenous formulations of combo nanocapsules could have facilitated varying speed and mechanism of uptake, which may impact antibody class-switching [44]. Size characterisation of the MS nanocapsules also indicated particle aggregation, which may have concentrated the amount of antigen taken up by APCs, resulting in greater humoral responses. These observations point to the ability to control size and structure of the silica template and resulting protein nanocapsules which may therefore serve as useful in tailoring nanocapsule vaccine formulations towards a desired immune response.

The most significant antibody response was seen from mice vaccinated with soluble UreA with TiterMax, while no significant response was seen from soluble UreA alone, as expected. However, consistent with previous reports that antibody is not protective in *H. pylori* infection, colonisation was not reduced in this group (Figure 5).

Analysis of gastric infiltrating lymphocytes in the stomach of experimental groups found the highest CD4^+^ T cell populations in the stomach tissue of mice vaccinated with combo nanocapsule (with and without TiterMax), followed by the MS nanocapsule with TiterMax and soluble UreA alone vaccination groups. The SL3261pYZ97 positive control group had the highest CD4^+^ T cell population levels overall, which was expected based on previous studies using this vaccine model [18,30]. Several studies have demonstrated the importance of infiltrating CD4^+^ T cells in successful protection against *H. pylori* infection [30,45]. The results in this study overall indicate that the combo nanocapsule formulations have the potential to generate strong local CD4^+^ T cell responses, which is indicative of a cellular-mediated response against *H. pylori* infection. However, in this experiment, this was not reflected in a reduction in colonisation for these groups. Possibly, the CD4^+^ infiltrate had not reached sufficient magnitude to elicit protection at the time point studied—further studies assessing cellular populations over time and antigen specificity would be required.

Investigation of the induction of gastric infiltrating CD8^+^ T cells found that mice vaccinated with soluble UreA without adjuvant vaccination induced the highest CD8^+^ T cell response, followed by combo nanocapsules with TiterMax and combo nanocapsules alone. CD8^+^ T cells have a critical role in the protection against intracellular bacterial infections. Despite their important role in the defence of mucosal tissue [46], CD8^+^ T cells are not considered crucial in the gastric immune response to the extra cellular *H. pylori* infection. However, a number of studies characterising CD4^+^ T cell responses to *H. pylori* infection have also reported significant infiltration of CD8^+^ T cells into stomach tissue [47,48,49], perhaps suggesting a role for CD8^+^ T cells in response to *H. pylori* infection.

Analysis of infiltrating CD11b^+^ inflammatory monocytes found that soluble UreA alone and combo nanocapsules alone generated the highest response, followed by combo nanocapsules with TiterMax and MS nanocapsule with TiterMax groups. The CD11b^+^ marker is commonly expressed on monocytes, natural killer cells, macrophages and neutrophils. The role of macrophages in *H. pylori* infection is thought to be in the exacerbation of gastritis [50], but it is unclear whether they play an essential role in vaccine-mediated protection against *H. pylori*.

Analysis of *H. pylori* burden in the stomach of vaccinated mice found that only the SC/MS nanocapsules with TiterMax vaccine was effective in significantly reducing *H. pylori* burden. Notably, the SC/MS nanocapsule with TiterMax vaccination did not induce significant antibody levels, suggesting that the SC/MS nanocapsules were able to confer a level of protection without a measurable humoral response. Recombinant proteins as vaccine antigens often elicit weak immune responses, and consequently are delivered alongside an adjuvant [51]. TiterMax Gold adjuvant was selected as the adjuvant for this study as a commercially available water-in-oil emulsion, with decreased tissue toxicity and antibody titre production comparable to other water-in-oil adjuvants such as Freund’s Complete Adjuvant [52]. Water-in-oil formulations induce rapid recruitment of lymphocytes and antigen-presenting cells (APCs) to the injection site [53], leading to exogenous antigen capture, processing and presentation, resulting in activation of T cells [54,55].

As discussed above, previous infection studies have found that antibody generation against *H. pylori* infection is poor and may not be necessary for successful protection against infection [41,56]. Protection against *H. pylori* infection is primarily cellular-mediated and associated with a number of immunological factors, including a strong CD4^+^ T cell response, functional leptin receptors and MHC class II [40,57,58]. The SC/MS nanocapsule with TiterMax group did not show significant levels of infiltrating CD4^+^ T cells, which is unexpected considering the significant reduction in *H. pylori* gastric burden seen in mice vaccinated with this formulation. Consequently, the results of this study warrant further investigation into the protective mechanisms of antigen nanocapsules against *H. pylori* infection.

To conclude, in this study, the efficacy of UreA-based nanocapsules as a vaccine model was tested in an in vivo *H. pylori* challenge model. Mice vaccinated with SC/MS nanocapsules with TiterMax were found to have significantly reduced stomach burdens of *H. pylori*. These results demonstrate that the nanocapsule vaccine model has potential to be an effective vaccine delivery system that can induce both humoral and cellular responses, depending on formulation, and warrants further investigation for efficacy against *H. pylori* and other pathogens.

## Figures and Tables

**Table 1:** Experimental groups used for the vaccine trial.

Group	Vaccine	Boost	Dose per Vaccination	Challenged
A	Salmonella positive control	-	10^7^ CFU	✓
B	PBS	PBS	100 µL PBS	✓
C	PBS	PBS	100 µL PBS	✗
D	TiterMax^®^ Gold Adjuvant only	TiterMax^®^ Gold Adjuvant only	100 µL PBS	✓
E	Soluble UreA with TiterMax^®^ Gold adjuvant	Soluble UreA with TiterMax^®^ Gold adjuvant	20 µg of antigen in 100 µL PBS	✓
F	Large nanocapsules with TiterMax^®^ Gold adjuvant	Large nanocapsules with TiterMax^®^ Gold adjuvant	20 µg of nanocapsule in 100 µL PBS	✓
G	Small nanocapsules with TiterMax^®^ Gold adjuvant	Small nanocapsules with TiterMax^®^ Gold adjuvant	20 µg of nanocapsule in 100 µL PBS	✓
H	Combination nanocapsules with TiterMax^®^ Gold adjuvant	Combination nanocapsules with TiterMax^®^ Gold adjuvant	10 µg of large nanocapsule + 10 µg of small nanocapsule in 100 µL PBS	✓
I	Soluble UreA without adjuvant	Soluble UreA without adjuvant	20 µg of antigen in 100 µL PBS	✓
J	Large nanocapsules without adjuvant	Large nanocapsules without adjuvant	20 µg of nanocapsule in 100 µL PBS	✓
K	Small nanocapsules without adjuvant	Small nanocapsules without adjuvant	20 µg of nanocapsule in 100 µL PBS	✓
L	Combination nanocapsules without adjuvant	Combination nanocapsules without adjuvant	10 µg of large nanocapsules + 10 µg of small nanocapsules in 100 µL PBS	✓

**Table 2:** *H. pylori* 16S primers for qPCR amplification [33].

FORWARD	5′-CTTAACCATAGAACTGCATTTGAAACTAC-3′
REVERSE	5′-GGTCGCCTTCGCAATGAGTA-3′
PROBE	5′-[FAM]TAC CTC TCC CAC ACT CT[TAMRA]-3′

## Data Availability

The data that support the findings of this study are in this published article and available from the corresponding author upon reasonable request.
